# Early-onset blount’s disease in a two-year-old child

**DOI:** 10.12669/pjms.42.(ICON26).15704

**Published:** 2026-04

**Authors:** Nida Asad, Nida Ilyas Shamsi

**Affiliations:** 1 Nida Asad, MCPS, MRCGP(INT), MHPE. Specialist, Department of Family Medicine, The Indus Hospital and Health network Karachi, Pakistan; 2 Nida Ilyas Shamsi, FCPS. MRCGP(INT), MHPE. Consultant, Department of Family Medicine, The Indus Hospital and Health network Karachi, Pakistan

**Keywords:** Blount’s disease, Bowing, Epiphysis

## Abstract

Blount’s disease is an acquired growth disorder involving the medial aspect of the proximal tibial physis, epiphysis, and metaphysis. Infantile Blount’s disease, one of its variants, commonly presents with progressive lower limb bowing and limb length discrepancy. Because physiological bowing and nutritional rickets are far more common in this age group, early-onset Blount’s disease is frequently overlooked in primary care settings. Early diagnosis is crucial, as timely intervention can halt disease progression, prevent worsening deformity, and reduce the need for surgical correction, whereas delayed recognition may lead to irreversible growth disturbances. This case is noteworthy because it highlights the importance of early clinical suspicion and radiological evaluation of pathological bowing in a toddler presenting first to a family medicine clinic. We report the case of a two years old girl with bilateral lower limb bowing, normal biochemical findings, and radiological features like bilateral medial tibial metaphyseal beaking (spur formation) with genu varum deformity and preserved bone density, findings consistent with early-onset Blount’s disease.

## INTRODUCTION

Determining the underlying cause of bowing is crucial, as conditions like Blount’s disease and rickets can appear similar but require different treatment approaches. Blount’s disease involves abnormal changes in the medial side of the proximal tibial physis, leading to significant varus deformity and medial rotation of the upper tibia. It is generally classified into three main types based on the age of onset and clinical features. The first, Infantile or Early-Onset Tibia Vara, typically affects children aged one to five years, with radiographic signs often becoming apparent between ages one and three. This form tends to worsen after the child begins walking and is commonly bilateral. The second, Juvenile Late-Onset, occurs in older children and is characterized by a later presentation compared to the infantile variant. The third, Adolescents with Late-Onset, may present during adolescence or later in life and can affect one or both legs, appearing unilaterally or bilaterally.^1^

The prevalence of Blount disease in the United States is <1%.^1^ Specific data regarding occurrence rates in the Asian context remain scarce, but existing literature suggests that the condition is less common. Blount’s disease, though rare, has been reported across several Asian countries through isolated case reports and small series. In India, a three-year-old girl was diagnosed with bilateral early-onset Blount’s disease managed conservatively.^2^ Chandankere et al. reported favorable outcomes in late-stage infantile Blount’s disease treated with acute single-stage medial hemi-plateau elevation combined with metaphyseal osteotomy. Their series demonstrated effective deformity correction and restoration of limb alignment in older children, supporting acute surgical correction as a viable option in advanced disease stages.^3^ Eighteen months old boy presented with the complaints of bilateral bowing of lower limb with normal biochemical investigation and radiological survey revealed Blount disease.^4^ From Pakistan, a case was also reported from Zhob, Baluchistan in 2022, where a three-year-old presented with bowing and diagnosed to have Blount’s disease.^5^

Collectively, these reports underscore the disease’s presence in Asia and the need for earlier recognition and better epidemiological tracking. More robust epidemiological studies are required to accurately ascertain the prevalence of Blount’s disease in these regions and to understand the nuances of this condition in diverse populations. Although African-American ancestry, obesity, and early walking are known to be risk factors for Blount’s disease, the exact pathophysiology of the illness is still unknown. From limb length disparities to articular cartilage abnormalities, the severity varies.^6^,^7^

The most immediate effect of Blount’s disease is the physical limitation it imposes. Children may experience difficulty walking, running, and participating in sports or activities that require agility. These physical challenges can lead to a more sedentary lifestyle, which in turn can increase the risk of obesity and other mental health-related issues as well. The frustration of not being able to keep up with peers can affect a child’s social interactions and lead to feelings of isolation. The age and severity of presentation determine the course of treatment for Blount’s disease, which ranges from bracing to surgery. Among the available treatment options are hemi-epiphysiodesis, corrected proximal tibial osteotomies with either acute or progressive fixation, and knee-ankle-foot orthoses (KAFOs). If left untreated, Blount’s disease may progress to severe and irreversible lower limb deformity, including worsening genu varum, limb length discrepancy, and abnormal tibial torsion. These structural abnormalities can result in chronic gait disturbance, persistent pain, and reduced functional capacity in adolescence and adulthood.

## CASE REPORT

A two years old girl was brought by her father to the family medicine clinic with a six-month history of progressive bilateral bowing of the lower limbs, noticed by her parents. The child had no history of systemic illness, and there were no associated symptoms such as fever, weight loss, lethargy, or reduced activity. There was no history of pain in the lower limbs or prior trauma.

She was born at term via normal vaginal delivery, and her immunizations were up to date. Developmentally, she achieved both sensory and motor milestones within the expected age range. She began walking at 11 months of age, slightly earlier than her siblings. The patient has two older siblings, aged seven and five years, both of whom are asymptomatic and have no history of lower limb deformities. There was no family history of similar complaints.

On examination, her weight was 12 kg, height 80 cm, with a body mass index of 18.75 kg/m². General physical examination revealed a normally shaped head with no facial dysmorphism. There were no clinical signs of rickets, including frontal bossing, rachitic rosary, wrist widening, or double malleoli. Bilateral bowing of the lower limbs was observed.

Her complete blood cell count was reported as: Hemoglobin: 9.3 g/dl, Total white blood cell count: 9.13 × 10^9^/L (neutrophil 38.7%, lymphocyte 45.5%, monocyte 6.1%) and platelet count: 397 × 10^9^/L. Serum urea, creatinine and electrolytes levels were with inthe normal range. The biochemical investigation revealed serum calcium 10mg/dl, alkaline phosphatase levels of 259 U/L and Vitamin D levels of 60.4 ng/ml.

**Fig.1:**
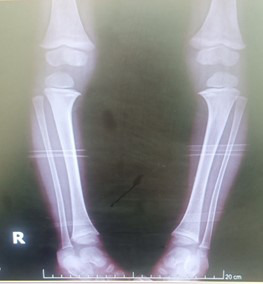
Anteroposterior radiograph of both lower limbs showing bilateral genu varum with medial tibial metaphyseal beaking (spur formation) and preservation of bone density, findings consistent with early-onset Blount’s disease.

Upon diagnosing the two-year-old girl with early-onset Blount’s disease, a comprehensive management plan was initiated by opting for conservative management with a knee-ankle-foot orthosis (KAFO), following current recommendations from the American Academy of Orthopaedic Surgeons (AAOS) and recent literature, which suggest bracing as the first-line intervention for young children with early-stage, progressive Blount’s disease. Observation alone was not considered appropriate in this case due to risk of deformity progression, which can lead to permanent angular deformities and gait abnormalities if left untreated. Unlike most reported cases that describe milder deformities or later presentations, this patient presented at a very young age with pronounced tibial varus, making early orthotic intervention critical. The use of KAFO in this context not only provides mechanical support while allowing some range of motion but also represents a proactive approach aimed at minimizing the need for future surgical correction, adding novelty to this case report.

The family received counseling regarding the importance of adherence to wearing the orthosis for the prescribed duration daily, usually ranging from 23 hours a day unless contraindicated for hygiene reasons. Regular follow-up appointments in pediatric rehabilitation department were scheduled to monitor the child’s progress.

In conjunction, a referral to a pediatric orthopedic specialist was arranged for further evaluation, particularly regarding potential future surgical interventions such as hemiepiphysiodesis, hemi-epiphysiodesis, or osteotomy if conservative measures fail. Success of KAFO treatment will be evaluated based on reduction in tibial varus angle, improvement in gait, and radiographic evidence of normal medial tibial growth.

## DISCUSSION

Bowing of the legs is a common presentation in children, particularly in lower-middle-income countries. Causes can be physiological, which typically resolve as the child grows, or pathological, resulting from underlying disorders that require intervention. Correct differentiation between these is essential, as untreated pathological bowing may worsen over time, while physiological bowing generally does not require treatment.

For primary care physicians, a systematic evaluation of a child with leg bowing is critical. Conditions such as Blount’s disease and nutritional rickets can present with similar clinical features but require different management approaches. In this case, the diagnosis of early-onset Blount’s disease was supported by its clinical features, normal biochemical parameters and radiological findings

Risk factors such as early ambulation, obesity, and use of walkers may exacerbate tension on the posteromedial proximal tibial physis, contributing to deformity development. Early detection allowed timely intervention with a knee-ankle-foot orthosis (KAFO), aiming to prevent progression and reduce the likelihood of future surgical correction.

By explicitly comparing clinical, biochemical, and radiological features to other common causes of leg bowing, this case highlights the importance of early identification and targeted management of Blount’s disease, particularly in children under three years of age with significant varus deformity.

It effects both legs in about 60 % of the cases. Blount’s disease was first reported in the year 1922 by Erlacher, describing involvement of the medial half of the proximal tibial epiphysis is generally regarded as an early description of what we now call Blount disease (tibia vara).Walter Blount later provided a more complete clinical, radiographic, and pathological characterization in the 1930s, and the condition was subsequently named after him.^8^ Infants with Blount’s disease exhibit sudden medial angulation “beaking” and bending of the medial cortical wall of the proximal tibial metaphysis on lower limb radiography. A posterior oriented protrusion at the proximal tibial metaphyseal level was seen on lateral knee radio-graphs.^9^

Physiologic bowing, congenital bowing, rickets, osteomyelitis, metaphyseal chondrodysplasia, Ollier disease, and traumatic deformity are some differential diagnoses for bowing of legs which are to be considered when seeing a patient with bowing of legs.^10^ It is indeed possible for a child to present with simultaneous conditions, such as vitamin D deficiency and structural deformities indicative of Blount’s disease. A nuanced understanding of laboratory results and their clinical correlation is paramount.

It is significantly important for family physicians to not hastily attribute bowing to rickets without a comprehensive evaluation, which includes detailed history-taking and a physical examination. Additionally, judicious ordering of tests, such as serum calcium, phosphate, alkaline phosphatase, vitamin D levels, and relevant imaging studies, can help differentiate between these conditions. Failure to accurately diagnose can significantly impact a child’s quality of life; untreated severe bowing may lead to pain, mobility issues, and social ramifications as the child grows.

The child’s age and the severity of Blount’s disease determine the treatment strategy. In cases of significant bowing before the age of three, a knee-ankle-foot (KAFO) or hip-knee-ankle-foot (HKAFO) orthosis is commonly prescribed. If the deformity is moderate to severe, or persists beyond the age of four to five, corrective surgery such as proximal tibial osteotomy may be indicated. Management of such cases requires a multidisciplinary approach, involving orthopedic surgeons, pediatricians, and physiotherapists, to ensure both physical correction and supportive care.

In addition to physical limitations, children with Blount’s disease may experience psychosocial and mental health challenges, including reduced self-esteem, social anxiety, and frustration due to mobility restrictions or visible limb deformity. Early intervention, both orthotic and surgical, along with supportive counseling, can help mitigate these psychosocial impacts and promote overall well-being.

### Informed consent:

Informed verbal consent was obtained from the child’s father prior to inclusion of clinical details in this case report, in accordance with ethical guidelines for human subjects.

### Authors’ Contributions:

**NA:** Conceptualized the case report, collected and interpreted clinical, biochemical, and radiological data, literature search and drafted the initial manuscript.

**NIS:** Assisted with literature review, critically revised the manuscript and contributed to discussion

Both the authors have read the final version and are responsible and accountable for the accuracy and integrity of the work.
